# An Account of the Fever Which Occurred among the Theological Students of Lane Seminary, during the Fall of 1842, and the Ensuing Winter

**Published:** 1843-11

**Authors:** Thomas Carroll

**Affiliations:** Cincinnati


### WESTERN JOURNAL

OF

### MEDICINE AND SURGERY

---

NOVEMBER, 1843.

---

Art. I.—*An Account of the Fever which occurred among the Theological Students of Lane Seminary, during the fall of* 1842, *and the ensuing winter.* By Thomas Carroll, M.D., of Cincinnati.

Before entering on the particular consideration of the fever which is the principal object of this paper, I shall make some observations about the locality in which the Lane Seminary is situated; and the mode of living adopted by the students, &c.

This institution is situated a mile and a half from the eastern border of Cincinnati, on a plateau elevated five hundred feet above the Ohio. This plateau stretches and gradually descends several miles to the eastward, where it meets the Little Miami river; on the south it is bordered by the

Ohio, and on the north-west by Mill-Creek. Towards the river it breaks into steep declivities; but in other directions, the descent is more gradual. Towards Mill-Creek it is broken into steep declivities or gently sloping hills. The geological formation from the river to the summit of the hills, is mostly of transition limestone, alternated with thin beds of shale; these are, however, so thin that they should be denominated seams. Water is obtained by digging to the depth of about thirty feet; but few wells, however, yield an abundant supply. Springs are occasionally met with, yielding reasonable supplies of good water. Currents of air which play over the surface must of necessity be pure, as the elevation insures an exemption from fogs or humidity.

It would seem from the foregoing considerations, that the location of the Seminary would insure as complete an exemption from disease, as that of any other; and, that no situation in the Mississippi Valley could offer a better prospect for health than it does. Indeed, if the experience obtained during the last forty years may be allowed to decide, there can be but little doubt of the truth of the proposition.

The habits of the students at the Lane Seminary, have probably differed but little from those of young men at other, or similar institutions. Their rooms are in a house which stands in an exposed situation, and is four stories high—has a large number of rooms, each of which is eleven or twelve feet long, and eight wide, lighted by one window, with the exception of those at the angles of the building, which have two, each. These rooms are warmed by open stoves, heated by wood. It has been the habit of the students to take care of their own rooms, which of course has not always been done in a way that a lady would have dictated. Most of them have lived at a common table, which has been furnished in a plain but substantial manner. Six or seven, however, boarded themselves during 1842, and most of these were Grahamites; indeed, all lived in an abstemious way. All with a single exception had the fever—he, I believe, ate animal food occasionally, and he was also too far advanced in life to be in much danger of the disease. Among these students arose the worst forms of the fever. But one student who had the disease boarded in a private family, and the form in which he had it was not severe. Six cases occurred in families who had admitted some of the sick students. There were two cases in the Rev. Mr. Goodman's family; these occurred in boys who, so far as was known, had not mixed with any of those who had the disease at the Seminary. All who had the fever, besides the students, were under thirteen years of age; and one was not more than two years old.

That there has existed in the Mississippi Valley, during many years, a fever whose characteristics have been different from the intermitting, remitting, synocha or synochus, is, I think, capable of the clearest demonstration. In an essay published in this Journal, in 1842, (Vid. *Western Journal*, vol. v., p. 20,) I described this fever as one of a continued type; and contented myself with detailing some of the leading features belonging to it, with the nature of the localities in which I had observed it. Names in the West, as well as in other places, have had too much influence in dictating the treatment of disease, and particularly of fevers that have had the fortune of being called typhus; I was, therefore, unwilling to misguide my readers by endeavoring to distinguish it by any other name, than that of continued; though, as will be seen, there can scarcely be a doubt, that it is identical with the New England typhus of Nathan Smith, or that called typhoid by more recent writers.

The first evidences of fever at Lane Seminary, were evinced in a young gentleman who had spent a few months in northern Indiana; and who was taken ill soon after his return, which was in September. His fever did not, in the opinion of Dr. Dodge, appear to be of the same kind as that which was afterwards developed among the students; he thinks it was a case of remitting fever, combined with dysentery. The next case was that of Mr. Hicks, who had been travelling in Illinois for some time, and had been at home about a week before he was attacked. A short time before his return he had been sick, but had recovered. He was attacked early in September, and was not well on the last of November. Dr. Dodge, who attended him, was first under the impression that he had nothing but a mild form of remitting fever; but the long continuance of the fever, and the symptoms attending the last stage, caused him to class it with those that followed. On the first of November another case occurred; on the fourth two more; and from that time until the twenty-seventh December, but few days passed without a new case. The number affected was thirty-two; twenty-four students and eight children.

At first, most of the cases began with slight indispositon, which generally continued but a few days; head-ache was a frequent symptom, and lasted for two or three days more; and then a chill of some hours duration completed the formation of the fever. During the whole forming stage, slight chilliness was experienced particularly at the approach of night. The skin was mostly dry, though there was in a few cases partial sweating, which generally occurred at night. This condition of the surface, however, did not last long; it became dry, and yielded to the touch that peculiar sensation which so generally belongs to typhoid fevers. The pulse was quickened and more resisting than natural, and there was occasionally slight nervous disturbance that sometimes continued either in an undiminished, or aggravated form, throughout the disease. The fever might now be said to be completely formed, and continued a longer or shorter time according to circumstances. A few recovered completely in two weeks, while others were down from twenty days to three months. Most of the patients were convalescent in twenty-eight days. Through the first ten days, the tongue was mostly slimy and covered with a white fur, which after some time became brown on the centre and back part. In a few cases the organ had a chapped appearance; in two or three only was it red; and in two cases dryness supervened. One or both of these conditions was present in two cases that proved fatal.

The urine in most cases was scanty and high colored, and in a few was voided with difficulty towards the termination of the disease. The face was frequently flushed, but some times little alteration could be observed; this symptom was however more frequent during the advanced stages, or after the first ten days. It often appeared day after day on but one cheek, and in the afternoon. It mostly preceded sweating a few days, but when this supervened, it subsided for some hours, and reappeared on the following afternoon. The pulse generally exceeded its natural frequency from ten to twenty strokes per minute. In some of the adults it was one hundred and forty, and in children occasionally more. It was generally regular, full, and round, without much tension; in a few cases it was irregular, vibratory, tense, and sometimes feeble, though mostly resisting. In two of the fatal cases, the pulse was regular, round, and not hard until near the last. The hearing was affected in but few patients; in three of the fatal cases it was imperfect. Towards the termination of the fever sweating was frequent, though not universal, and had generally the effect of mitigating the fever; indeed, in many cases, it seemed to be the mode by which the disease left the system.

The understanding remained clear for the most part; yet there were six patients in whom delirium supervened at some period of the fever. Slight wandering occurred in others, and was present in a greater or less degree, in all the cases that terminated fatally, though not through the whole course of the disease.

Sudamina appeared in six patients only, and in but one of those that terminated fatally; but the lenticular spots, considered essential to the typhoid fever of Louis, Chomel, Bartlett, and others, appeared in a well marked form in but two cases. Both of these were children, one eight, the other four years of age. It is true, that there was in a few cases an eruption, but it was not of the kind that is considered essential to typhoid fever.

There was either diarrhœa, or very easily excited bowels, in a large number of cases; indeed there were but few exceptions. The discharges per anum were generally of good color, but not consistent; and hæmorrhage, so much to be dreaded, occurred in two cases only, which will be noticed by and by. Meteorism, and gurgling on pressure, were present in most of the cases; the former, in particular, to a greater or less extent in nearly all. This condition is not always brought about by unassisted morbid action, but, in my humble opinion, by the mode of treatment, both as regards medicine and diet. Soreness and pressure over the region of the ileo-cœcal valve, could be discovered in most cases; and sometimes there was diffused tenderness over every part of the abdomen, though this symptom was by no means common. In a few patients there was irritation of the stomach, and medicine was retained with difficulty: this organ, however, mostly bore with ease whatever was taken. Most of the patients had considerable thirst, and desired cold drinks. Abdominal pains were not unfrequently present, and in a few cases were severe; bleeding from the nose was not an uncommon occurrence towards the close of the fever.

With these general observations, I shall proceed to detail the mode of treatment adopted in the management of this disease. So various have been the methods pursued by different practitioners in the treatment of what is called typhus fever, that he who prescribes for it must render himself obnoxious to the censure of those who have adopted the different views of different authors, or practitioners; this will not, however, deter me from being careful in detailing the method pursued in the treatment of the disease under consideration.

I did not see any of the cases until the 25th November; after that period, I attended all of them in conjunction with Dr. Dodge, until the 27th January, when the Dr. was seized with the same disease. I then attended alone until it disappeared in his case, about the first of April. Anterior to the time I was called, Dr. D. had attended all the cases, but occasionally had the advice of Drs. Mussey and Worcester.

Dr. Dodge and myself usually made alternate visits, and compared notes every evening, so that the treatment was conducted with a perfect understanding of what each had done; thus a very steady course was pursued throughout.

One of our first objects was to see that the patients had apartments sufficiently large and well ventilated; and that their bed and body linen should be frequently changed. In accomplishing these objects we experienced but little difficulty, as a number of those who fell sick were kindly invited to the houses of the professors, or wealthy neighbors, where every friendly office was promptly and cheerfully performed. Those students who were not sick became nurses in turn, so that proper attention was paid, both by day and night. It may be added, that as all the adult patients had well cultivated minds, and had arrived at definite conclusions as to the prospects of a future state, calmness, without unnecessary despondency, was manifested in every case, so far as I know.

When at the outset we found our patient with a strong, hard, and frequent pulse, or indeed when the frequency was but little if any above the natural standard, with accompanying head-ache, and flushed face, we bled to approaching syncope; immediately after, the warm dash was used, or sometimes warm sponging, and in a few cases the cold dash was administered either over the whole surface, or on the head alone. The next object was to purge freely by giving from ten to fifteen grains of calomel, and following it in half an hour or more with a free dose of salts, jalap, or oil. I was, myself, favorable to the use of jalap, and therefore gave it, in preference to the other articles. After thus reducing the system, it was agreed to direct the warm shower or sponging every few hours during the first several days; and afterwards less frequently. I felt anxious that all the patients should take tartrate of antimony in minute doses, throughout the disease, until the approach of convalescence or sinking; if these irritated the bowels or stomach, opiates were to be added, or used to prevent it; and in those cases in which the diarrhœa was difficult to control, the antimony was to be discontinued. Occasional small doses of calomel were thought proper; where there was irritation of the bowels the blue pill was substituted in its stead.

In prescribing mercurials it was not our object to affect the salivary glands, though a gentle action we did not fear; we had little or no confidence in its removing the fever, however much it might mitigate the symptoms. When diarrhœa should appear, it was our purpose to allay it by Dover's powder, soda, &c. Aperients were to be used when admissible, but no active purging should be induced after the first few days of the fever; one, two, or three evacuations daily, it was thought would be sufficient. My own predilections led me to favor the use of jalap, or epsom salts for this purpose. Dr. Dodge preferred salts, while Professor Mussey had a very decided opinion in favor of rhubarb and soda combined; in cases of debility this combination had a good effect. Often no cathartic medicine was administered for days in succession. Emetics we occasionally tried, and found them beneficial in several cases; in one, however, vomiting had an unfavorable effect. Probably no certain rule can be laid down to govern the use of emetics in fever; and in those of a remitting or intermitting type, they not unfrequently seem to act disadvantageously; in continued fevers, however, they operate with more advantage.

When the abdomen was in a meteoric state, with or without tenderness, or the bowels painful, fomentations of various kinds were prescribed, with dry-cupping, blistering, &c.; scarifications with cupping, were resorted to in a few cases. For pain in the head, or more serious affection of it, cold applications to the scalp with blistering, &c., were resorted to; the latter was of much service, the former more problematical. It may be proper to observe that in cases where bleeding was resorted to more than once, the patients were not benefited by the second operation; indeed I am opposed to bleeding after the first week, except in a few rare cases. I do not recollect that I have ever known a patient to recover, who was largely bled at a late period. Whatever brings the pulse below the natural standard in point of strength, has had, so far as I have observed, an unfavorable effect. At the outset we concluded it was only proper to moderate the force of the morbid action, rather than to attempt to cut it short; as we considered the disease in a great measure of a self-determined character.

It will be admitted that in a disease where there exists local lesions likely to destroy life, and where the accompanying fever is of a high grade, whatever would lessen its force would benefit, or prevent the progress of those lesions; and by this means enable the system to overcome the fatal tendency that otherwise would attend such lesions. Hence the benefit of moderating the force of the circulation in this fever. Advantage would then be derived from various means, as bloodletting, laxatives, diaphoretics, &c. But of all the means within the reach of the profession none seems to me equal to the tartrate of antimony, when given in such way that the stomach shall not be much disturbed by it, yet that the circulation shall be brought under its control, which will be known by the pulse becoming less frequent, less hard, and less constricted, or more natural in its action. Small doses will, when continued during several days, gradually bring the action of the heart and arteries under its influence, so that the frequency of the pulse will be lessened two strokes or more per minute, at the same time that all the other symptoms will be mitigated—the skin will be more natural, the heat more regularly diffused, head-ache, if it exist, will be moderated, and the various secretions more normal.

Not a few authors and distinguished professional men have given their opinion in favor of the use of antimony in continued fevers. I am happy to say that among these are Professors Drake and Dudley.

The following cases will illustrate my views with regard to the use of this medicine in the treatment of the fever under consideration; and of the advantage that may be derived from bloodletting under certain circumstances.

Mr. Hildreth, son of the distinguished S. P. Hildreth, M. D., of Marietta, was taken sick on the 4th November; was chilly for three days anterior to confinement to his room. At the close of this period the fever was completely developed. There was head-ache, thirst, a dry skin, and restlessness. To relieve these symptoms, he took six grains calomel, six of aloes, and six of rhubarb, which acted well; on the following day, I believe, he took by the advice of Dr. Worcester, five grains blue-mass, and some castor-oil, which also acted. The fever, however, continued, with intense head-ache; this the patient thought could be relieved by bleeding, and he got one of the students, who was a physician, to open a vein; thirty oz. were drawn by which he experienced immediate relief. Before being bled he had occasionally used cold applications to his head; during the time of their application, there was no pain, but he had oppression of the chest with pain and difficulty of breathing, which was relieved by the removal of the cold from the head, when the pain became re-established in that part One or more other cases, as will be seen, were affected in the same way, by the same treatment.

For some time he took a combination of spirit. nitr. dul. with occasional doses of rhubarb and blue-pill. I first saw this patient on the 25th November; his pulse was then one hundred per minute; his fever constant, and skin dry and hot. He had sudamina in great numbers about the chest. One-tenth grain of tartar-emetic, was ordered every four hours, with occasional doses of rhubarb, and five grains of the blue-mass every other night, or even more seldom than that. He was directed the warm shower every four hours, or when the shower was not convenient, sponging was admitted. After this course was instituted, the pulse but seldom exceeded ninety beats per minute. The cause of this alteration I ascribe to the influence of the antimony in a great measure, though the external use of water had no doubt a happy effect.

Mr. Tyson, aged about twenty-five years, was seized with head-ache, which occasionally remitted. It was mostly in the back part of the head, and was accompanied with some degree of stupor; slight chilly sensations were also complained of. On the fourth day of his illness I found him with intense head-ache, and a strong pulse, ninety per minute. The tongue was loaded with a white fur; yet he complained but little of his stomach or bowels; he, however, had considerable thirst. As this gentleman was naturally robust, I concluded that an active plan of treatment would be the best; I therefore bled him to the fainting point. After this I fixed his head over the edge of the bed and separated his hair so that the scalp could be easily reached by water, one gallon of which was slowly poured from some height on his head. I directed that if head-ache returned it should be repeated; this was, however, unnecessary, as there was no recurrence. Very soon after bleeding, fifteen grains of calomel, and thirty of jalap were given, which acted freely. At my next visit I found my patient sitting up and with very little fever. I directed him to abstain from bodily or mental exercise, to live on bread and water for some days, and occasionally to take laxative doses of salts. His fever altogether subsided at the end of ten days from the first evidence of disease.

Mr. Kinney, of about the same age as Mr. Tyson, and with a fine constitution, was taken very nearly in the same way. He had been sick three days before I saw him. I directed the shower bath, and prescribed fifteen grains of calomel, and thirty of jalap. After the cathartic had operated, he was bled, agreeably to my instructions in case head-ache supervened. By this course the patient was relieved of all unfavorable symptoms in a few days.

Mr. Ford, a young man of great physical power, and of regular habits, was attacked in nearly the same way as the last two gentlemen. I first drew thirty ounces of blood, and directed an active cathartic of calomel and jalap, with an occasional warm shower, one however, was directed immediately after the bleeding. This course so effectually subdued the fever at the outset, that it never returned with any violence. He took during a week or more the tenth of a grain of tartrate of antimony every four hours. In a short time after the beginning of the treatment, sweating commenced, mostly at night, and within a few days seemed to carry off the fever.

It is now my melancholy task to lay before the reader an account of a few of the cases in which bleeding was either not resorted to at all, or at a late period. I shall confine myself to the description of five cases, four of which terminated fatally, the remaining one recovered with much difficulty.

Mr. Olney was taken sick on the 1st December. His health had been good, and he was among the most successful students of the Seminary. His diet had been for months exclusively vegetable, or nearly so—though so far as I know, no evidence of debility had been observed as induced by his mode of living. For some time before he was taken, he had occasionally watched with Kidder, whose case will be noticed by and by; but I believe he had not been much exhausted from that cause. Before he was confined to his room he had been unwell during several days. His head had ached, and he had felt indisposed to action. Some hours before I saw him, he had become chilly, and the pain of the head had increased—his skin had become more dry, and his hearing, which had hitherto been but slightly affected, had now become much impaired. When I saw him he complained of noise in the head, with considerable difficulty of breathing. His face was flushed and hotter than other parts of the body. The pulse was one hundred and twenty, full and hard, generally regular, but when he moved it became quicker and less harmonious. He had but little pain, save in his head, though his bowels were not altogether easy, as he complained of fulness and slight soreness on pressure. His tongue was covered with a white fur, but was not dry. Viewing the case as one fraught with great danger, I proposed bleeding, although, as yet, none of the patients had been bled; my patient would however, not submit to the operation. This opposition grew out of a predilection that he had for the "steam system" of practice; and on similar grounds he would not take calomel. Under these circumstances I probably ought not to have made any prescription; yet the anxiety felt to save the life of a promising young man, overcame my professional pride. I directed a cold shower every eight hours, with salts, and minute doses of antimony. The cold water gave him considerable relief, after each application, during the first two or three days, but ceased to have much influence afterwards. Dr. Worcester saw the patient on the following day, and made no other prescription; on the ensuing day Dr. Dodge saw him, and found his head still much affected; delirium had supervened some hours before his visit; he opened the temporal artery and drew a considerable amount of blood, which for a few hours seemed to render the patient more calm. This condition gave way to delirium, &c. I again saw the patient at the end of seventy-two hours from my first visit. I found him sitting up in bed, and wanting to get out; his eye was wild and watery; his countenance sunk, and rather pale, with much subsultus. This last symptom had been present from an early period, as I observed slight twitchings of the tendons when I first saw him. There was also at this time sudamina over the neck and chest, but no lenticular spots. Diarrhœa was now established, and there was fulness of the abdomen; but from the condition of his mind I could not determine as to its tenderness. Combined with these symptoms was constant watching, which, indeed, had existed from the first. Dr. Dodge, on the preceding day, had given one or two ten-grain doses of calomel, which had operated. I directed a repetition, with a blister to the head, which was, however, not applied until the following evening. I did not again see him until forty-eight hours had elapsed. Dr. Dodge in the mean time visited him, and had directed more calomel in doses of ten-grains, which he took. The symptoms had all been getting worse; he was lying in a constant state of irritation, or motion from nervous disturbance. His tongue was tremulous, chapped, and brown, and his breathing difficult, &c. At 9 o'clock of the same evening he expired.

Nineteen hours after death, Dr. Dodge and myself proceeded to examine the body. It had been my opinion from the beginning, that the brain was deeply implicated, and that it had continued in a diseased condition (or at least its membranes) until death. I had indeed given it as my opinion that the direct cause of death lay in the brain. Dr. Dodge, however, was inclined to think that the lesions of typhoid, as described by Louis, had caused a fatal termination. Under these impressions we began the dissection.

The tunica arachnoidea was adherent to the pia-mater throughout, and opake to a considerable extent. The pia-mater was vascular beyond what is usual; and the brain being cut into, exhibited red points through its substance; there was, however, but little effusion. These appearances satisfied me of the sudden termination of the case in death; my friend, however, had his attention fixed on the abdomen as the seat of the leading cause. We found the stomach but little altered, nor did we find much, if any, disease, along the line of the upper bowels, until we arrived within six inches of the ileo-cæcal valve. From this point until we reached the cæcum, we found an unusual number of elliptical plates, and they presented the following appearances: The edges were raised above the surrounding mucous membrane, from one, to two or three lines; most of them, I think, were elevated not less than two lines: these elevated edges were ragged, and rose nearly perpendicularly from the surface of the bowel, on their outer edges, but descended more gently towards the centre of the plates; and in one or two places, a small extent of the mucous membrane was removed by ulceration. The extent of this ulceration did not exceed a quarter of an inch in one place, and I believe there were but two places in which it extended through the mucous membrane. That part of the mesentery connected with the diseased bowel, was more vascular than natural, and had a red or venous color. The mesenteric glands in this portion were enlarged and much more solid than natural, though they had not a dark color when cut into, but on the contrary were whitish; this circumstance led me to regard them as in a tubercular condition. The spleen was increased in size, and much diminished in solidity; it broke down too easily under the finger. The liver was healthy, and the kidneys were apparently in the same condition. We found no disease in the lungs; nor had there been any pleuritic affection.

A question now remains whether bleeding would have saved the life of this patient? That his life would have been prolonged by it, I have no doubt, because free bleeding at the onset would have materially influenced the morbid action in the brain, and very possibly have altogether relieved that organ, and its membranes, from whatever congestion or inflammation then existed: and I am unable to see why it would not have had a most salutary influence on the inflammatory action going on in the elliptical plates, mesentery, and its glands. If, indeed, the mesenteric glands were tuberculated no treatment would probably have had much influence on them; yet, they might have remained slightly diseased for years, without doing much injury, as but few of them were in a morbid condition.

Mr. Kidder, aged about twenty-six years, was taken ill on the 4th November. He was located in the third story of the college edifice, in a room that was much exposed to changes of the weather, having one large window opening to the south, and another to the north. The casing of these windows not being well secured, the air was freely admitted at all times; and in cold or cool weather the room was uncomfortable, even with fire.

This gentleman's habits had been, for some years, those of close and laborious application to study; and his diet strictly vegetable with the exception of a little milk occasionally. Brown bread, potatoes, apples, and water, constituted most of his food. For some years past he had worn flannel, but during the few months preceding his illness he had substituted cotton for it. Two or three days before his illness, he had walked several miles, and returned on the preceding day through rain. He then felt unwell, could not get his room warm, or at least he thought so. On the ensuing day, he was found in his room, complaining of cold, with head-ache, and debility. He was removed to Dr. Stowe's, in whose family he had every attention conferred that humanity could dictate. His apartment was large, and well ventilated; and he was constantly attended either by Professor Stowe, or by one or more of the students, until his death.

I did not see him during the first three weeks of his illness; but he was regularly visited by Dr. Dodge, who had the occasional advice of Drs. Mussey and Worcester. He had constantly taken laxatives or purgatives; and calomel, camphor, and ipecacuanha, as often as thought necessary. He had also been occasionally sponged with warm water. When I first saw this patient I thought him very ill, and so expressed myself to Dr. Stowe. I feared that he would be long sick, and that he would probably not recover. His pulse was from one hundred and twenty to one hundred and thirty per minute; the hearing difficult; the tongue covered with a white fur; the skin mostly dry; the countenance flushed, particularly during the after part of the day; and the eyes had a started appearance; his mind had occasionally wandered, and he was restless, with inclination to stupor.

We directed the further efforts to the mitigation of the fever, or force of reaction, which we endeavored to do by having the body sponged with warm water every few hours, and by giving minute doses of tartrate of antimony every two or four hours: he took the eighth of a grain at a dose; and five grains of the blue-mass were directed every twelve or twenty-four hours, with rhubarb, and, sometimes, with the addition of soda. This course was continued during twenty days, when we flattered ourselves that our patient was doing well. His bowels had been easily moved, and no pain, nor much soreness, or tenderness to the touch had been manifested; slight meteorism had, however, existed from the beginning. But on the 10th December, the meteoric condition of the bowels suddenly increased, with tenderness and increased gurgling on pressure. In the neighborhood of the anus a swelling was found to exist, which extended over the left hip. From the 10th to the 15th, these symptoms increased, with a more laborious respiration, and less regularity of the pulse; when, on the last named day, a slough was observed anterior to the anus, and on the right side of the perineum: this slough increased until it was two inches in diameter; on the 16th, it suddenly burst open, and a large amount of fæces was discharged through the opening. This singular circumstance of course placed our patient in a dangerous condition. From this time, for many days, fæcal matter combined with pus continued to drain from the opening. His urine also seemed to pass without control, and the tenderness and fulness of the abdomen increased, until the 22d, when to the already unfavorable symptoms, was added hæmorrhage of the bowels; a large amount of blood was evacuated, which seemed to come from a high point of the intestines, and, as afterwards appeared, from the ileum.

To prevent instant death, laudanum and wine were administered in large quantities. To render the patient comfortable, his linen was changed, and his person washed twice a day, until the termination of the case. Soon after the bleeding from the bowels, the extremities of the left side became paralyzed, and continued unaltered until about the 12th of January, when a gradual improvement began; but this improvement was limited, and he did not regain the use of either extremity to any considerable degree. About the same time that paralysis occurred, the left eye inflamed, and an abscess formed in the brow, which suppurated slowly, and when opened discharged matter of a strumous character.

On the second day after the hæmorrhage, beef-tea was given the patient, which he continued to take for some time in considerable quantities, with farinaceous food of various kinds: bread and milk, with potatoes, formed his chief diet.

During most of the time after the formation of the slough, he took four grains of quinine daily, in divided doses; laudanum was given uninterruptedly, until the last, with wine in greater or less quantities—to the extent of a pint daily for some time. The diarrhœa, which had been constant from the time the slough took place, moderated about the 17th. On the 5th January, there was cough, and severe pain in the right side; these continued unabated until the 10th; on the 22d they were much mitigated, and eventually nearly subsided. Towards the termination of the fourth week of his disease, inflamed spots of a circular character appeared, mostly on the abdomen and back; very soon after, pimples or pustules formed, at first filled with a serous fluid, which soon assumed the appearance of purulent matter: these pimples broke in a few days, leaving a small excavation, sometimes a line or more broad; both before and after they discharged matter, they had more the appearance of those produced by tartar-emetic ointment, than any thing else that I now recollect. Deep ulcerations proceeded from some of these spots, and several of them became the source of abscesses, both on the back and abdomen, which, when opened, contained a drachm of matter each; those on the back became the cause of unfavorable bed-sores. On the sacrum a few of these eruptive spots terminated in an extensive sore, about the first of January. The right trochanter or hip, from pressure alone, exhibited a deep slough very soon after. The alkaline poultice seemed to be the best application in removing all the sloughs. To relieve his bed-sores we procured a hydrostatic-bed; this had a most happy effect, and seemed to be the means of prolonging the life of the patient for days. I cannot too warmly recommend this kind of bed to the attention of the profession.

The swelling of the left hip had remained after the sloughing of the perineum for some days, in an undiminished state, when Dr. Dodge made an opening into it, about two inches from the anus, and withdrew a piece of cellular membrane, of considerable extent; fæces with pus were evacuated at the same opening, and to a considerable amount. A few days after, a small opening took place near the left trochanter through which pus and fæces were also discharged. These occurrences decided that the abscess had a connection with the rectum; we will see by-and-by the nature of the connection.

On the 22d January the patient had much pain in the abdomen, and in the lower part of the rectum, with more flatulence than usual. As he had taken no purgative from the time the perineal slough occurred, I conceived that the fæces had accumulated in a solid form, so that the rectum had not power to discharge them; upon examination I found this to be the case. Injections of warm water were administered, which, by a little manual interference, relieved him of the accumulated mass. I directed a medium dose of castor-oil, which carried off a large amount of fæces. On the 25th, Dr. D. directed more oil, which again acted well. A month had now elapsed since our patient had begun to improve, and notwithstanding the various unfavorable symptoms, his appetite had been good, and he had improved in strength and even in flesh. Profuse and frequently repeated sweating had occurred, which had lessened the force of the circulation; the pulse was less frequent and more regular. But about this time an evident change began to take place for the worse. The sore on the trochanter had enlarged, and had mostly thrown off the dead parts, yet the granulations now seemed to cease filling up the cavity, and those of the perineal sore by the 2d February had become flabby, and much diminished. The skin over the gluteus maximus was disposed to slough. I made an incision several inches long, through the integuments, which brought to view the bare fibre of the maximus. On the 13th, it was evident that his system was about yielding to the disease. I saw him on the 15th, and in a few minutes he breathed his last.

Twenty hours after death I proceeded to examine the body, assisted by Drs. Lawson and Warder. The friends of Mr. Kidder were unwilling that we should open the brain, which

I regretted, as the cause of the paralysis lay no doubt in that organ, or its membranes; we, however, examined carefully the spinal marrow, and the contents of the abdomen and chest.

On opening the abdomen we found the vascularity of the mesentery but slightly more than natural; and the glands appeared in a healthy condition. By looking on the ileum from without, we perceived a number of bluish spots that afterwards proved to be connected with Brunner's glands; these spots were not more than the fourth of an inch in diameter. The spleen and liver appeared healthy, and were smaller than usual in a subject so large; indeed all the abdominal viscera seemed attenuated; probably his meagre way of living had brought this about. Little or no evidence of disease was found in the stomach. In tracing the mucous surface downwards, the valvulæ conniventes were found thickened and contracted; but the inflammation that produced this condition had disappeared, as there was neither redness, nor congestion of the vessels. Peyers' glands could be perceived with difficulty, and all that were discovered seemed to be in a healthy condition; the isolated follicles, however, had been inflamed, though they had nearly regained their wonted condition. Two ulcerations were found in the head of the colon: one, a half inch long, by a third wide, not yet cicatrized; a portion of the mucous membrane nearly thrown off, still floated in the cavity of the bowel, connected with the ulcer by a slender attachment; the other ulceration had completely cicatrized.

The left lung adhered, though not very firmly, throughout its inferior and external surface to the pleura costalis, and considerable fluid was effused, but not amounting to more than a few ounces. The inferior lobe of the left lung, and the superior of the right, were rendered partially impervious to air by tubercles, some of which were as large as a hazelnut—they had not yet arrived at the stage of softening. This tubercular state of the lungs would in all probability have caused death in a few months.

The pelvic viscera were found in the following condition: That part connecting the bladder with the parietes of the abdomen was inflamed, and had yellowish serum effused in its cells; the cavity of the bladder was so much diminished that it would not have contained more than a gill of fluid. The fundus and posterior part of the bladder, down nearly to the entrance of the ureters, was inflamed, and the walls were three-eighths of an inch thick. On the mucous surface there was no disease in the inferior part, but above the entrance of the ureters, it was thickened and corrugated. This condition of so important an organ, would, of itself, have rendered life miserable. The mucous coat of the rectum was thickened and much congested; about an inch and a-half above the anus, on the left side, was an opening through which the fæces had passed; this orifice would admit the end of the finger with ease. It descended along the lateral and inferior side of the rectum, and eventually opened on the right side of the raphe of the perineum, where the first slough took place; but before it arrived at this point, it gave off a branch, which terminated in the great cavity over the gluteus maximus. This muscle throughout its whole extent was completely bare. The cavity extended some distance beneath the fascia of the thigh; it was nine or ten inches long, and reached from the perineum to the trochanter; and contained during most of the time pus combined with fæces. It is my opinion, that this cavity first discharged its contents into the rectum, and that, afterwards, the fæces were pressed into it. We examined the spinal cord through about a foot of its length, beginning at the last of the cervical vertebræ, and descending along the dorsal: we were unable to discover any disease.

The two following cases occurred in the family of the Rev. E. Goodman: Henry, the elder of the two, was twelve years old, and Moses nine. Henry had apparently a good constitution; but the younger had for a long time been occasionally affected by chest diseases; this was attributed to his simulating the diseases of his mother, who has long labored under a disease of the lung; indeed the feebleness of her constitution made us early fear the result of the cases under consideration.

The elder boy was taken on the 23d December, with chills, pain in the head, and dullness of hearing, and was confined to bed at once. His father administered thoroughwort, &c., and on the 24th he had an alvine evacuation; from this time his bowels remained easily moved, until near the termination of his illness. The pulse was one hundred, or one hundred and ten, regular, and had considerable strength, when I first saw him, which was on the sixth, or seventh day of his illness. Dr. Dodge had seen him once or twice before. At the beginning, his tongue had a white coat, which continued until the 27th, when it became red down the middle, and afterwards over the whole surface. When this was discovered Dr. D. was called in; antimony was directed in doses of one-tenth of a grain, every few hours, and blue-mass, rhubarb, and soda, so given as to produce two or three evacuations daily. The warm shower-bath was also directed, from one to three times a day. This course was steadily pursued until the 16th day; during this period his pulse gradually abated until it was only ninety per minute. Sometime during the night of this day, and a little after having had a discharge from his bowels, he complained of severe pain in the abdomen. I saw him on the following day, and found his breathing laborious, his abdomen swollen, and tender to the touch, accompanied with frequent disposition to vomit. Although his bowels had been somewhat tender and flatulent, yet it was obvious, that some circumstance of a most serious kind had given rise to these symptoms, as it was now evident that peritoneal inflammation had commenced, and that the case was beyond the control of medicine. I prescribed morphine, and minute portions of calomel, which were to be repeated occasionally, with fomentations, until the next visit. On the next day, the violence of the pain had yielded, but the case appeared equally hopeless. Dry cupping was resorted to with some advantage.

Late in the evening a consultation was held with Dr. Mussey; little, however, resulted from it, as nothing more of importance was attempted. Before the consultation, a few small doses of cathartic medicine had been administered, but after developments showed that the prescription was not judicious. His mouth, which had been sore from the beginning, was now worse; and the head-ache that had also existed from the commencement, now was more intense. At the end of twenty days from the time he had been taken, death supervened.

Dr. Dodge and myself examined the body eighteen hours after death. On cutting into the abdomen we found considerable lymph effused, which in the lower part of the cavity was mixed with a quantity of fæcal matter, that had escaped from an ulceration in the ilium. The bowels were extensively glued together. A portion of the ileum, six inches distant from its termination, was congested and of a venous color. That part of the mesentery connected with it was deeply tinged with blood. Some of the mesenteric glands were diseased, and enlarged, and one contained about a drachm of badly digested pus. This abscess would no doubt have burst into the cavity of the peritoneum, had death not supervened so early. We found the spleen enlarged, of a bluish color, and the substance easily broken down; but the liver and kidneys were healthy in appearance. The mucous surface of the stomach was healthy, unless that portion most nearly connected with the spleen had too much vascularity; but as we traversed the mucous surface of the ileum, we discovered deep discoloration of it, through a space of about fourteen inches, corresponding with the external discoloration. Almost in the centre of this congested part was the perforation of the bowel. This perforation would have admitted a common quill. Its edges were not elevated, and the edge of the muscular coat was black. There was no evidence that there had been a gland where the perforation took place, neither did we find, any where in the diseased surface, the appearance of elliptical plates or isolated follicles. But below this congested part, in a space of six inches, which reached to the ilio-cœcal valve, these plates were numerous: they seemed altogether healthy. Their folds could be distinctly seen through the mucous membrane, yet they had not in the least elevated it. The lungs were in a morbid condition. There were no lenticular spots, nor sudamina on the skin, nor had any been observed during the boy's illness.

Moses, the younger boy, was taken sick on the 31st December, after having endured much fatigue through the day, in acting as carrier of the Watchman of the Valley, for his father. He had head-ache, and pain in the bowels, for a day or two before he was taken down. On the 1st of January, he took a blue-pill which acted freely. During two or three days after the 1st, his mind constantly wandered. In the first few days there was but little done; I did not see him until the watching had terminated in sleep, which continued almost constantly for several days. A very mild course, consisting of laxatives and warm shower-baths, &c., was pursued for several days, or until the end of the tenth day. There appeared to be nothing unfavorable in his pulse or its action; his respiration was laborious, and quick, though he did not complain of pain in the chest. Meteorism had existed to some extent from the beginning, but it now increased. On the 15th Dr. Mussey saw him in consultation with us. He was cupped, and stimulants were directed; his reason from this time wandered, and he succumbed on the 18th.

On examining the body sixteen hours after death, we found the following appearances: Spleen enlarged and easily broken down. The peritoneum in a healthy condition; the mesenteric glands healthy; the mesentery but little, if at all, congested or vascular. But on traversing the mucous surface of the alimentary canal, we found through the space of three feet, the elliptical plates all ulcerated; their edges not raised, but the ulceration had removed the plates down to the muscular coat. There was very little discoloration of the mucous membrane, and the ulcerated as well as surrounding parts were pale. No unhealthy appearances existed in the liver

Lymph and serum to the amount of six or eight ounces were found in the right pleura; and the inferior lobe of the right lung was carnified, and the whole lung congested. The evidences of disease were much less marked in the left lung, as there was but little solidification of its substance, and the serum only amounted to two or three ounces.

Mr. Maxwell, a young gentleman of rather slender make, and about twenty-five years of age, who had for some time, probably years, abstained from animal food almost entirely, was attacked with fever on the 2d November. He had been unwell for several days previous, but had paid little attention to it; on this day, however, he took oil. On the 4th, Dr. Dodge directed another dose of oil, which operated on the 5th. Head-ache had existed from the first, but it became constant on the 7th November, and on the 9th he was removed from his room to Professor Allen's, in whose family he received the most humane attention until his recovery. On the 12th the head-ache was unabated, and was indeed worse; it was thought, although the fever had existed ten days, that bleeding might be of service; a vein was opened, but fainting almost immediately ensued, a circumstance that mostly takes place in the advanced stages of this fever. But little advantage was derived from the operation. During the second and third week of his disease, applications of cold water were made to the head; they had the effect of relieving it when the cold was intense, but when his head was easy, he always felt fulness and oppression of the chest; this might possibly have been the cause of palpitation of the heart, of which he complained much during the first three weeks. His pulse had, however, been irregular from the beginning, and generally ranged from one hundred and thirty to one hundred and forty per minute throughout his illness.

I first saw this patient on the 25th November. I then found most of the foregoing symptoms existing. His mouth was covered with a viscid mucus, and was slightly sore. The bowels had been sufficiently open, and had even been disposed to diarrhœa; there was meteorism with tenderness to the touch over the abdominal parietes. Although he had taken some calomel, I directed ten grains of blue-mass every four hours, until the bowels should be moved, and a blister was applied to the back of the neck, by which complete relief was obtained. In two or three days after I saw him, ptyalism occurred, which evidently had a happy effect. He gradually improved for about a week. His appetite began to return, and he ate two oysters with a little other nourishment. Twelve hours after, his appetite craved more, and another oyster was taken. His stomach refused to digest them, and although when they were eaten he was able to sit up, he suddenly relapsed, and in three days his abdomen became greatly distended, and tender; delirium soon supervened, and continued without intermission during four weeks. His pulse again beat one hundred and thirty per minute, was irregular, and sometimes intermittent. We first gave oil, which acted pretty freely; and he occasionally, afterwards, took rhubarb and soda, with oil sometimes. Very soon after this relapse Dr. George Hildreth, of Marietta, arrived on a visit to his brother, whose case was first described, and who was still confined to bed. In consultation about Maxwell's case, he proposed that antimonials should be given in minute doses for the purpose of regulating the action of the heart. We assented to this suggestion, and enjoined the Dr. to watch the effects of the medicine. I believe that sickness was once produced by the antimony, and he vomited bile. This eventually had a favorable influence on the circulatory system. Injections were used for about a week, as occasion required. Blistering over the abdomen was also resorted to, and with advantage. At the end of a week the abdominal irritation was much mitigated, and by the 1st January, he was much better. He now slowly improved, and by the middle of February, he thought himself able to go down stairs; but about this time he ate one or two green apples, which produced colic in a few hours; this, however, was soon relieved. The pulse, even at this time, was irregular, and rapid. I now understand that it is more natural, and that this young gentleman enjoys good health.

Mr. Hogshead, was seized with the fever on the 21st of November. Dr. Dodge, I think, saw him on the 22d, and gave ten grains calomel, with scammony, &c.; this medicine had been repeated before I saw him, which I think was on the 21st February. Evacuations had not been obtained; his mouth was inflamed; he spit considerably; and his pulse was one hundred and twenty per minute, full, and resisting, but regular. The surface was warm, the head ached, and he was restless. He had not been bled, but warm sponging had been resorted to with advantage. I directed an emetic of ipecacuanha, which operated moderately; this was followed by purgatives, which acted pretty well. This patient was throughout his sickness more constipated than any of the others. He generally slept badly, unless under the influence of opium. On the 10th day of the fever, he suddenly took a severe pain in the left side of the chest; and very soon after he expectorated pretty large quantities of blood, slightly mixed with mucous. A severe cough began with this condition of the lungs, and continued throughout the remainder of the disease; it received a check, however, on the 15th January, by the sudden discharge of about a quart of mucus and pus, which took place within a few hours; this gave him immedidiate relief from the most distressing symptoms. Anterior to this, the cough had been very severe; for many days he had thrown up rust-colored matter, which had been expectorated with difficulty, and was accompanied with severe pain, and with a respiration of thirty-six per minute; the pulse was one hundred and forty, or one hundred and fifty at times. During the early period of his disease, he suffered from a burning sensation in the pit of the stomach, but this symptom yielded after some days. Emaciation rapidly followed the beginning of the fever, and continued until he was unusually reduced; this circumstance caused much trouble during the latter part of the disease, as bed-sores supervened, which were painful, and caused much irritation.

So soon as the lungs became affected, we began the use of antimonials, gum-arabic, and morphine, with the tincture of sanguinaria. The antimony, however, with laudanum, or morphine, was the chief remedy; the fifth of a grain of tartrate of antimony was steadily given every two or four hours, during many days, and opiates were administered at night in free doses, and sometimes were given every six hours. As the patient's bowels were constipated, oil, salts, or jalap, were given daily, or nearly so. Blisters were repeatedly applied over the chest, and poultices of bread and milk were often used to relieve pain, which they seldom failed to do. By pursuing a steady course, our patient eventually survived, and was completely convalescent at the end of three months from the beginning of the fever. After the case had progressed for some time, we regretted that we had not drawn blood, as we thought that the violent action of the heart would have been subdued by it, and that the inflammation of the left lung would have been prevented.

Hæmorrhage from the bowels may at all times be considered a grave symptom, and one that not unfrequently occurs in this fever. A case has already been detailed in which this symptom was manifested; that case did not terminate fatally from the effects of hæmorrhage, nor from lesions which had given origin to it; but from other and very different causes. The treatment adopted to prevent the recurrence of bleeding in that case, has been hitherto successful in my hands when hæmorrhage has occurred in typhoid fever. I therefore feel anxious to impress on the reader's mind a clear idea of the mode of treatment. Anterior to 1834, I had never known a case of this fever recover in which hæmorrhage had taken place; and I had uniformly found that daily purgation had been advised and pursued. This fact led me to the conclusion, that purgative medicines should not be given in such cases. I accordingly determined that I would in the treatment of a number of cases, should they occur to me, avoid all laxative medicines, and that I would keep the peristaltic action quiet through a number of days. This determination I have carried out in the treatment of a considerable number of cases during the last nine years, and I have not as yet been disappointed. The following case I will detail, in order to illustrate my views of the method of treatment that should be adopted in hæmorrhage of the bowels, as well as for considerations growing out of the nature of the disease.

Dr. Dodge, of Cincinnati, aged thirty-five years, had attended the patients at the Lane Seminary, from the commencement of the fever. Most of the time he had visited the establishment every other day; this attention, combined with his other professional duties, rendered his business laborious. He was unwell for several weeks before he was confined to bed; but on the 14th January, he began to have restless nights with occasional head-ache, accompanied with a sense of fulness of the abdomen. He was constipated, and his appetite was variable. During this time he attended to his professional duties with his usual industry, but took aperients occasionally, with alteratives mostly composed of the blue-mass. About a week before he was confined, he bled himself, but not freely, as he was soon disposed to faint. On the 27th January, he was first confined to his room, and on the 28th I was called to see him. I found his pulse hard and full, but not very frequent, as his natural pulse is not over sixty per minute; there was restlessness and vigilance; the skin was dry; there was head-ache; his tongue was covered with a white fur. Under all these circumstances, I proposed to bleed him, to which he did not agree; I then directed an emetic of ipecacuanha, which acted mildly, and brought on free prespiration, which lasted for several hours. To keep up this action three grains of calomel, and ten of Dover's powder, were given, which had the effect through the night of the 28th. On the 29th, he took salts, which acted well. On the 30th, he sat up, and left his room for a few hours; but on the 31st, his head-ache returned, and in the afternoon, I bled him to the fainting point, and showered him with warm water. The combined influence of these means, very much subdued the fever through the night, and on the morning of the 31st, I gave him the third of a grain of morphine, to relieve the purging and the restlessness. The day was spent comfortably, but not without fever; in the evening he took the eighth of a grain of morphine, and at 2 P. M., of the 1st February, the eighth of a grain more. He had a tranquil day, but the night was spent with fever. In the morning of the 2d, he took one and a half grains of calomel, and eight of jalap; this small dose acted several times. The following night was spent pretty well. I saw him however, at five A. M., and found him with considerable fever. I concluded to begin the use of antimonials, but my patient neither then, nor afterwards, was willing to continue their use long—owing to this prejudice, they were not persevered in with constancy; he, however, took the eighth of a grain of tartrate of antimony every four hours; but two or three doses were taken before sickness supervened. His pulse became soft, and was reduced in frequency. Up to the 6th, his bowels were kept open with small doses of calomel and jalap, which answered the purpose, as his bowels were now very easily moved. Minute doses of antimony were occasionally given during this time, to moderate the fever; and morphine was taken to produce sleep. His tongue still kept well shaped, and was covered with a white fur; his cheeks began to be flushed in the afternoon, particularly the right cheek. His pulse during this time ranged from seventy-two to seventy-eight.

About the 4th or 5th, Professor Drake saw the patient, and proposed cupping over the right side of the abdomen. This advice was founded on the fact, that pain was felt on firm pressure on that part; but the suggestion was not acted on for several days; not because it was not proper, but for the reason that other applications (fomentations of various kinds) had been used occasionally from the beginning, and had given relief when uneasiness was experienced. Very few days had elapsed before I regretted that I had not insisted on the use of cups when they had been proposed by the Professor, for on the 8th more serious symptoms manifested themselves. After the 6th, the patient's bowels were moved with more difficulty, and there was more desire to have evacuations. On the 8th, he took early in the day, ten grains rhubarb, with one grain of calomel; and at noon four grains of jalap, and one grain of calomel; also, five grains of blue-mass, with an injection, which brought away bloody serum, with some small clots. It was obvious that considerable hæmorrhage had taken place, as the force of the pulse was much diminished. Immediately on the discharge of the clots and serum, I gave the sixth of a grain of morphine. A few hours after, the pulse was seventy-two, round and full. At 11 o'clock of the 9th, his bowels acted freely, no doubt from the effects of the medicine taken on the 8th. This evacuation contained considerable blood; the pulse was much reduced in power, and a little in frequency; the surface was cool, and without much perspiration. At 5 o'clock, A. M., of the 10th, I saw him, and found that the morphine taken after the evacuation, had kept him easy; his face was flushed, and at ten A. M., he complained of pain in the region of the ileo-cœcal valve, with soreness. The pain was unremitted, and continued so for some time. I put dry cups over the abdomen, had him scarified and cupped, and fomentations were constantly used; he was eventually blistered, and the oil of turpentine was used as a rubefacient: which means relieved the pain and tenderness on pressure. The meteorism, although existing from the beginning, now became worse and more distressing. Up to the 13th, no blood nor other matter, had passed, from the bowels. He had taken on the morning of the 12th, without my consent, ℈i. rhubarb, and fifteen grains blue-mass, which operated pretty freely on the evening of the same day, but the evacuation contained no blood; this was soon followed by another, which contained a gill. Morphine, camphor, and ipecacuanha, were given, and the morphine was continued with more regularity after the last hæmorrhage than it had been. On the 15th, his fever was less, pulse soft and regular, and the soreness of the side was mitigated; on the 16th the bowels were still more easy, and perspiration had extended further over the body. The amount of pain and abdominal soreness experienced in this case, may be conceived, when it is stated, that he was unable to lie on the left side, and only partially on the right; he was also unable to move himself, and whenever he was moved, he suffered severe pain.

From the 16th to the 24th, the soreness gradually subsided, and he had no bleeding. He took camphor, morphine, and ipecacuanha, up to the 18th, when the camphor was laid aside, and the others continued. Little or no other medicine was given during this period; indeed, my object was to avoid as much as possible alvine dejections. On the 17th, the tongue became brown, on the centre and back parts, which continued to the 24th. From the time that his bowels first became painful, he had difficulty in voiding urine for many days, as the muscles of the abdomen seemed not to be under the control of the will, and probably the bladder was in the same situation; he passed urine, however, about once every twenty-four hours. Through this period of eight days the pulse was almost constantly seventy-two per minute.

From the 24th, to the 29th, the symptoms gradually abated; the tongue began to clean on the 27th, and was left brown. No purgatives were given, but the bowels occasionally acted. Morphine, ipecacuanha, and small quantities of blue-pill, were alone given, and these promoted or seemed to promote perspiration. The occasional exacerbations were moderated by warm sponging. On the 1st March, he ate a small quantity of food, which was the first that he had taken during thirty days. By the 2d March, he was convalescent. A little restlessness followed the suspension of the use of the morphine, but he was no longer subjected to diarrhœa, to which, as has been seen, there was a disposition throughout most of the disease; he had now to take some mild laxatives to regulate the action of the bowels. His improvement continued until the 15th, when the tongue became sore, and the bronchial tubes were clogged. A cough supervened, which was most severe during the evenings. The mucous surface of the alimentary canal was also involved. This condition, I think, was probably caused by a too free use of condiments, calf's-foot-jelly, &c. His pulse now rose to ninety or ninety-five; this condition lasted for ten days, during which there was much irritation and debility; the skin was unusually dry, and there was constant vigilance, unless under the use of morphine. To render the skin more soft, he was rubbed all over with *fine* oil: this gave the greatest relief imaginable during the succeeding twenty-four hours, and after a few days, it was repeated, but not, I believe with the same beneficial effects. I had long wished for a case in which the external application of oil might be tried, as I believed, that in the latter stages of typhoid fever, it would be found useful; and the trial made with it in the present case, has satisfied me that many cases of fever would be mitigated by occasional application of oil over the whole surface. On the 25th March, convalescence was again established, and by the 1st of May, the Dr. was able to attend to his professional business.

During the illness of this gentleman, I had the occasional counsel of a number of the professional men in this city, but more particularly that of Professors Mussey and Harrison, which, during several days of the doctor's illness, gave me much consolation.

Before closing this paper, it is deemed proper to consider the relation in which the fever that has just been described stands to that which has usually been called typhus or typhoid fever, of New England and other parts of the United States, as well as of Europe, &c. It is known to the profession, that Professor Bartlett has written a work which extensively treats of the difference between what is called the typhus of the British, and the typhoid of the French; and has labored to show that the typhus of Nathan Smith, or of New England, is identical with what the French call typhoid fever.

We are not left in suspense about the diagnosis of this fever, for the Professor, with Louis, Chomel, &c., admits that the typhoid fever has characteristics which never vary, or in other words, that "in all cases of typhoid fever there is lesion of the small intestines. This lesion is peculiar, it is found in no other disease." Fevers not showing after death this lesion cannot be considered typhoid. Dr. Bartlett thinks we have a much less definite knowledge of the lesions peculiar to other diseases than of this.

Now the fever at the Lane Seminary appeared mostly among the students, and was confined within a very limited space; one case seemed to grow out of another, all having in many respects great similarity; yet the morbid lesions were not entirely the same, as related to the small intestines. I have reasons to induce me to believe, that a number of of those who recovered had no serious lesions, if any, of the small intestines; I will, however, adduce one case to demonstrate this, which will go to show, that there could have been little more than congestion of the mucous surface of the ileum, anterior to a certain period in the fever.

Mr. Lindsey, aged about twenty-three, had waited on Kidder for some days; and had not obtained his usual amount of sleep. He complained of being unwell—of dullness, and occasional sweating, with loss of appetite, &c. I advised him to cease from bodily exertion, and to take medicine. He located himself in comfortable lodgings, and was seen by Dr. Worcester, and then by Dr. Dodge. Four or five days after he began regularly to take medicine, I saw him. He then had a meteoric condition of the bowels, which were slightly tender to the touch; and his tongue was smooth and red. The pulse ranged from ninety to more than one hundred per minute, and was full and resisting. The bowels were moved easily—externally there were no lenticular spots, but he had sudamina. His fever progressed for about four weeks, and was treated in the usual way. From the rather more than usual fulness of the abdomen, Dr. Dodge and myself feared that there existed extensive ulceration of the elliptical plates, but an unexpected circumstance occurred, which determined that no such condition was present. We found our patient without meteorism, soreness or tenderness of the abdomen; but we found both the parotid glands sore, hard, and somewhat swelled. This inflammation of these glands went on through a number of days. The swelling threatened to terminate in suppuration. As it advanced the face became involved in it, and the eyes were for a short time nearly closed. Fomentations were used, and free leeching, with blisters, by which, and other means, the patient was relieved without suppuration, and rapidly recovered his health. This change took place about four weeks from the beginning.

The question now is, whether this patient could have had ulceration of Peyer's glands, anterior to the swelling of the parotids. The very sudden translation of the local affection from the abdomen to these glands, proves to my satisfaction that no ulceration did exist; for had it existed no cure could have taken place in so short a time.

But we will refer to the lesions in those cases that proved fatal, and see whether they agree with the notions of those writers who have taken so decided a stand on the question of certain lesions being essential to typhoid fever. In the case of Olney, the elliptical plates were diseased in the manner laid down by Louis, as essential to this form of fever; but the mesenteric glands were not affected in the way that this distinguished pathologist thinks necessary to typhoid; for instead of being rose-colored and softened, what few of them were at all diseased, were white in the centre, and much harder than usual. He had no lenticular spots, nor sudamina. Death resulted in this case not from the condition of the abdominal viscera, but from that of the brain and its membranes.

In the two cases that occurred in Mr. Goodman's family, it has been observed that the lesions were different. Neither of the patients had lenticular rose-colored spots, nor sudamina; but one had ulceration of Peyer's glands; yet the mesenteric glands were almost, if not altogether, free from disease. In the other the elliptical plates were healthy, but there was a congested or inflamed mucous surface in a small part of the ileum, and that part of the mesentery connected with it, and several of the glands were affected in the manner Louis thinks essential to typhoid. Kidder's case seems to demonstrate that the elliptical plates really had never been diseased; but that the isolated follicles had been inflamed, though not ulcerated; here also, the mesenteric glands were found apparently healthy.

These actual conditions of the lesions as they appeared after death, seem to decide, that this fever, although it had many of the characteristics of the typhoid fever of the French, &c., still wanted some of the essential elements of that fever; for Bartlett says, that the lesion of Peyer's glands is peculiar to typhus fever, and is found in no other disease. This assertion, if founded in truth, places the Lane Seminary fever in a singular situation; as some of the cases had no diseased condition of the elliptical plates and some had. The patients had lived together in the same house, before they were taken ill, and most of them eat at the same table. The disease seemed in all other respects alike, but our author would, according to his theory, divide it into different species. That this was not the case, I have not a doubt, because the experience I have had in similar fevers, during the past twenty years, assures me that a fever of this grade does exist, and does not at all times exhibit those symptoms, or autopsic developments, which are made essential in the typhoid of Louis. Even admitting that Louis has discovered a real distinction in the typhus fevers of a vast metropolis like Paris, it is not very likely that an identical fever would be found in a country so sparsely populated as this, and where the habits of the people are so widely different from those of the cities of France.

I regret that Professor Bartlett should have labored on this point so much, with so little prospect of fixing the true diagnosis of this subdivision of typhus fevers, or of the continued or winter fevers of the United States. Indeed, if the description of the French typhoid fever, as given by Chomel be considered correct, I am inclined to the opinion that the description does not altogether suit the winter fevers of this country. He says that out of one hundred and twelve cases, seventy-three came on suddenly. This statement seems to show, that our typhoid fevers are different in their mode of attack; for although we occasionally meet with patients who had been taken down within a few hours, yet in a very large number of those whom I have attended, or seen, the disease was slowly developed, a number of days often elapsing before the patients were confined to bed. This condition was known by loss of appetite, unusual disposition to sleep, or the reverse, with lassitude, &c. The case of Dr. Dodge will go to show the truth of this; he had fulness of the abdomen, restless nights, with some head-ache, and irregular action of the skin for two or three weeks before he declined business. I found in almost all the cases at Walnut-Hills, that several days had elapsed before the patient would acknowledge himself seriously ill; and when at last confined to bed, he could hardly tell how he had got to that situation; for frequently the chills were so light, that the patients were at a loss to know whether they actually were chills, or whether the cold sensation did not proceed from some external cause. That the winter fevers of this country always exhibit the lenticular spots, and mostly sudamina, I do not believe. If lenticular, rose-colored spots, had always existed in those cases which at various times have fallen under my care, I think I should have observed them; sudamina certainly very often occur in bad cases, but they not unfrequently appear in other diseases. I am aware that Louis has seen cases in which the patient had walked about for a week or two, and some of them even attended to their ordinary business during that time; this is not unfrequently the case in this country, and in my opinion is one of the strongest evidences of a close relation between the two fevers.

None of these symptoms are, however, uniformly manifested in every case. The eruptions accompanying these fevers I regard as accidental, or owing to circumstances belonging to individual cases; and I have but little doubt, that they appear more frequently in Europe, than in this country, and more often in cities than in other localities; which, if so, I suppose is owing to the inferior quality of the food and clothing of the people of Europe, and of cities, to that of those in the country. I make this remark with regard to the poor.

Professor Bartlett labors with diligence to demonstrate the characteristics of typhoid fever, but when he comes to the treatment, he is much less decisive; and although he has had an experience of fifteen years in it, he does not come to any direct conclusion, as to the mode of treatment that should be adopted; he even thinks that no treatment has had as much good effect, as any or most of the methods that have been pursued by different practitioners. When a distinguished *medical man* pays, through a considerable period, much attention to a disease, it might at least be expected, that he would come to a decision as to the remediate means that should be adopted. But in the present case, we are disappointed; and I cannot avoid dissenting from the Professor, as I believe the typhoid fever can be very considerably controlled by medicine, and that favorably.

That many have lost their lives by the rash treatment of practitioners, I acknowledge; but such practice is founded upon false premises. A belief in the cutting short of fevers by a herculean method of treatment, has caused not a few practitioners to do too much in continued fevers of a typhoid character. This is particularly the case in the West, but in the East they possibly have erred upon the ground of debility, and have long thought stimulants too important in the treatment of these fevers. When this fever is rightly understood, the mode of treatment can be so regulated that much benefit will result. If we reflect, that there is mostly, if not always, a lesion of the small intestines, more or less extensive, as well as of the mesentery, and its glands; often of the lungs, brain, or its membranes; we will at least come to the conclusion that a mitigation of these local affections may be brought about; and if this can be done, the fever wears itself out, without producing any mortal lesion. But Dr. Bartlett thinks that the cause of this fever exists in the condition of the blood; this notion may be correct, very possibly is, yet we often find patients getting well; and I think more often under some methods of treatment than others, or where none has been adopted.

It may not be improper to consider a little further the relation in which the disease of which we are speaking stands to those of a similar character, that have appeared in the United States, and particularly in the Mississippi valley. It is now nearly thirty years since my attention was first drawn to the continued fevers of the West; this was in the winter and spring of 1814. The fever occurred in Columbiana county, Ohio, where I then resided. A few of the cases, I believe, lingered through the summer of that year. Although I had not at that time even thought of studying medicine, I had ample opportunities to examine with care a number of cases which I waited on. This fever was at that time thought to be the same disease that had a short time before prevailed in New England, and in other Eastern districts of our country, as well as in Canada. In the year of which I speak, I believe it extended, in Eastern Ohio, from Cleaveland as far South as Marietta; and was more general and grave in its character than it has since been in the same district of country. Some of the patients whom I knew, had the disease from one week, to two or three months: in all those cases in which it ran its course early, death was the result.

I saw no more of this disease until years had rolled away. In 1823, sixteen cases occurred on an elevated point, two or three miles south-east of the town of New Lisbon. Several of these cases I saw in consultation; and I found them treated with bark, wine, calomel, emetics, blisters, and corrosive sublimate mixed with lard, and rubbed on different parts of the body. Cathartics might be added with other articles. Of those who submitted to this treatment, but three or four survived, and eight died. One poor fellow had resisted the doctors, and had taken a little salts occasionally, up to the time I saw him—I said to him, *aside*, that he had better continue the same course, which he did, and recovered. The above disease appeared late in the fall, and was considerably scattered over the southern part of the county, which is bounded on one side by the Ohio, and on the other by Little Beaver creek, and is hilly and *thin*. During the same year but earlier, intermitting and remitting fevers prevailed extensively, on the low grounds near New Lisbon, and on Beaver creek, but was nearly unknown on the high grounds around and has been so at all times.

At various periods, since that time, I have observed and treated a continued fever, that mostly occurred during the winter; but sometimes, and not unfrequently, began towards the middle or latter part of autumn, and continued through the winter. This circumstance in its history has caused physicians (as I think) to class it with remitting fevers, at least this error has frequently taken place within my knowledge. It has, however, more often been called congestive fever, particularly in Illinois, Indiana, and other western districts. Since my removal from Eastern Ohio, I have heard of its being there called cold plague.

Probably there are but few points in our profession more difficult than, when this fever begins in autumn, or in the early part of the season, to decide whether it be remitting or typhoid. The typhoid, however, is mostly ushered in very slowly, has a peculiar condition of the skin, and is more continued in its character; it has the tendency to spread in a family where it begins, not by a sudden impression made at once, but by slow degrees, seizing the family and friends of the sick, one by one; indeed, all the cases in the same neighborhood can be frequently traced to a single one, as a nucleus. This was particularly the case at Walnut Hills. Mr. Kidder's case seemed peculiarly productive of others. Months sometimes elapses before this tendency to spread has an end.

One of the most conclusive evidences of its character, that

I know of, is its location. North of forty-three degrees of latitude, in North America, I presume this is almost the only fever which exists, aside from the erruptive and inflammatory kinds; and very possibly, in these northern regions, altitude has but little to do with its existence, or non-existence. Indeed I have been informed that winter fevers in Canada are those which are most dreaded. But when we get further South, we meet with remitting and intermitting fevers in certain localities: these localities are mostly alluvial in their character, though sometimes extensive plateaus are subjected to them. This is mostly the case when there is stagnant water on considerable portions of the surface. An exuberance of decaying and declining vegetable matter seems to be mostly essential to the propagation of these fevers. Persons living in high situations sometimes take remitting, or intermitting fever, merely by making occasional visits to districts subject to it. Singular circumstances occasionally occur; individuals living in localities wholly exempt from those fevers, may spend some days in a malarious district, during autumn, and no evidence of disease will be manifested until the ensuing spring, when they will be seized with the remitting or intermitting fever.

But as I have said the typhoid fever exists south of forty-three degrees; it is limited to a narrow space, and is mostly, if not altogether, confined to those situations in which the remitting and intermitting fevers do not occur. These localities, are most of the elevated table-lands of the Mississippi valley, and all the more rugged country through which the Ohio and its tributaries flow, with other like localities. That part of the country lying south-east of the Ohio, corresponds more particularly to this description. I do not recollect to have ever known the typhoid fever begin and spread on alluvions, but I have often witnessed it in the contiguous regions.

If I be not mistaken, this fever has occasionally occurred on the more elevated prairies of the West. Many of these prairies, I think, are situated from two, to two hundred and fifty feet above the rivers that exist in those regions. I travelled six hundred miles in Illinois, during the fall of 1835, and found that on the high prairies winter-fevers had been not unfrequent. Indiana has also suffered at many points with a similar disease. It has several times, I am informed, visited Richmond in that State; and during the past winter I heard of it as existing on the high lands within a few miles of Lawrenceburgh. From all I can learn, Ohio was visited by it at various points during the past winter and preceding fall.

Of the remote or exciting causes of this fever in our own country, nothing as yet is definitely known. So far as my own observation has extended, I feel well assured that the geological construction of the country has much to do with its origin; yet very different combinations of substratum at different times are visited by it. I refer the reader to some observations made by me, on this subject, in this Journal, vol. v., p. 20.

The mode of living has no doubt much to do with the origin of typhoid fever; yet I have known it to originate in very cleanly families, but seldom among those who lived well as to diet, and who were in the habit of eating animal food. This may, however, more frequently occur than I am aware of. The only fatal cases among the students, at the Lane Seminary, took place among those who had for a considerable time abstained from animal food, and all who were Grahamites had the fever. The following paragraphs are from a letter written by one of these gentlemen, Mr. H. E. Peck: I transcribe them for the benefit of those in favor of the vegetable plan of living.

As soon as he arrived at the Lane Seminary, "I began" he says, "to reduce my diet and curtail my hours of sleep—had previously lived upon a mixed, generous diet, and slept from seven to nine hours per diem; left off tea and coffee soon after my arrival here, next erased butter from my bill of fare, and finally gave up meat; lived chiefly on Graham bread, potatoes, baked apples and milk, and allowed myself between six and seven hours sleep daily, and every morning upon rising took a cold shower-bath. Found soon after I commenced reducing my diet, that I was growing weaker, and that my stomach was losing its tone—therefore adopted a vigorous system of exercise at wood-chopping; but instead of improving my health, labor seemed to diminish my muscular energy, and enervate my stomach, which at last became so *fractious*, that it would not bear any, even of the mildest food, without souring. My stomach was in this condition when I began to be troubled with an icy coldness of my feet, and occasional pains in my head. These symptoms continued for one or two weeks, when I was seized with pains especially in my limbs, and in the small of my back, and with a severe head-ache." These symptoms were soon followed by fever, with a pulse one hundred or one hundred and ten, &c. His fever lasted only fifteen days, and was of a medium character as to severity. He was treated by mild purgatives with occasional mercurials—and tartar-emetic in minute doses. During the first part of his convalescence, his stomach seemed in a good condition, but of a sudden, whatever he took soured; as yet he had taken little but vegetable food. Dr. Dodge ordered a change of diet, of which animal matter was to constitute a considerable portion. "This change," says Mr. Peck, "seemed to have a very beneficial effect on my stomach, which has since entirely recovered its wonted tone; I think my health is now better than it has been for three years." This letter was written a few weeks after his recovery, and now, in June, I believe his health still continues good.

In my early boyhood, a settlement of Highland Scotch was made in the neighborhood of my father's residence. These people endured great fatigue in making improvements in a new country; and added to their other hardships, was a megre diet, chiefly vegetable. The early pioneers of the country remonstrated with them, against their mode of living. The remonstrance, however, had no effect; and in about a year afterwards a slow fever broke out among them, I think early in the spring of 1807, and continued during a number of months—many of them fell victims to the disease. Its progress was slow, death only taking place after the lapse of several weeks.

That the hardships and abstemious diet of this people brought on the fever, I have little doubt, as those who lived in the same neighborhood and pursued a different manner of living, were not subject to it.

The location of the neighborhood in which this fever occurred, is near the Ohio river, in this State, and near its eastern border. The soil is thin, and the altitude equal to that of any other point in the State.

*June*, 1843.

